# The Use and Interpretation of Sodium Concentrations in Casual (Spot) Urine Collections for Population Surveillance and Partitioning of Dietary Iodine Intake Sources

**DOI:** 10.3390/nu9010007

**Published:** 2016-12-23

**Authors:** Joel Conkle, Frits van der Haar

**Affiliations:** 1 Nutrition and Health Sciences, Emory University, Atlanta, GA 30340, USA; 2 Emory University and Iodine Global Network, Atlanta, GA 30340, USA; fvander@emory.edu

**Keywords:** sodium, salt, iodine, UNaC, UIC, concentration, excretion, casual, spot, urinary

## Abstract

In 2013, the World Health Organization (WHO) called for joint surveillance of population salt and iodine intakes using urinary analysis. 24-h urine collection is considered the gold standard for salt intake assessment, but there is an emerging consensus that casual urine sampling can provide comparable information for population-level surveillance. Our review covers the use of the urinary sodium concentration (UNaC) and the urinary iodine concentration (UIC) from casual urine samples to estimate salt intakes and to partition the sources of iodine intakes. We reviewed literature on 24-h urinary sodium excretion (UNaE) and UNaC and documented the use of UNaC for national salt intake monitoring. We combined information from our review of urinary sodium with evidence on urinary iodine to assess the appropriateness of partitioning methods currently being adapted for cross-sectional survey analyses. At least nine countries are using casual urine collection for surveillance of population salt intakes; all these countries used single samples. Time trend analyses indicate that single UNaC can be used for monitoring changes in mean salt intakes. However; single UNaC suffers the same limitation as single UNaE; i.e., an estimate of the proportion excess salt intake can be biased due to high individual variability. There is evidence, albeit limited, that repeat UNaC sampling has good agreement at the population level with repeat UNaE collections; thus permitting an unbiased estimate of the proportion of excess salt intake. High variability of UIC and UNaC in single urine samples may also bias the estimates of dietary iodine intake sources. Our review concludes that repeated collection, in a sub-sample of individuals, of casual UNaC data would provide an immediate practical approach for routine monitoring of salt intake, because it overcomes the bias in estimates of excess salt intake. Thus we recommend more survey research to expand the evidence-base on predicted-UNaE from repeat casual UNaC sampling. We also conclude that the methodology for partitioning the sources of iodine intake based on the combination of UIC and UNaC measurements in casual urine samples can be improved by repeat collections of casual data; which helps to reduce regression dilution bias. We recommend more survey research to determine the effect of regression dilution bias and circadian rhythms on the partitioning of dietary iodine intake sources.

## 1. Introduction

In 2013, the World Health Organization (WHO) and partners convened a technical meeting to discuss reducing salt intake while using salt as a vehicle for iodine fortification [1]. The meeting concluded that there were a number of areas where these strategies could work together, including “cross-disciplinary research” and “shared surveillance of salt and iodine intakes through urinary analysis” [1]. The goal of the present review was to explore the use of casual urine sampling in the joint monitoring of salt intake reduction and iodine fortification strategies. 

Urine collections during a timed, consecutive 24-h period are considered the gold standard for the assessment of salt consumption in individuals and populations. The collection and handling of urine voids for 24-h is burdensome in a field survey setting, particularly when resources are constrained. Casual, or “spot”, urine samples can be obtained more readily and with less cost during the single-contact sessions typical of field surveys, but there are limitations on their appropriate use in the assessments and classification of salt intakes. 

The first objective of this report was to document the current methods and uses of data on urinary sodium concentration (UNaC) with the aim to determine if and how UNaC may be useful as a proxy indicator for salt intake at aggregate levels (e.g., populations and countries). The second objective of the report was to contribute to the development of methods for using concurrent sodium and iodine concentration (UIC) data from casual urine samples in the analysis and monitoring of salt iodization strategies. Of particular interest for Universal Salt Iodization (USI) monitoring is the determination of population iodine intakes derived from the three key dietary sources of iodine provision, i.e., the “native” dietary iodine obtained from the common diet, the iodine derived from iodized salt used in commercial food manufacturing and the iodine intake from iodized salt use in the households.

## 2. Materials and Methods 

For the first objective we searched the websites of organizations involved in setting global and USI standards and reviewed guidance from these organizations, providing an overview of recommendations for salt intake and documenting the rationale for considering 24-h UNaE the gold standard for salt intake assessment. We documented current approaches in monitoring salt intake by reviewing survey reports from nine countries using UNaC for national monitoring. The countries were identified primarily from previous systematic reviews. We identified one additional country from a basic internet search. For the nine countries, we analyzed UNaC methods, and we used data from three countries to assess the use of UNaC data in a time trend analysis. To inform objectives 1 and 2, we carried out literature reviews on sodium metabolism, and studies on agreement between UNaE and UNaC data. 

For objective 2, we carried out a literature review on UIC. We combined information from our review of UNaC with evidence on UIC to assess the appropriateness of methods to partition the iodine intake sources. We considered partitioning methods used by Haldimann et al. [2] that are currently being adapted for use in cross-sectional survey analyses, and focused on how UNaC and UIC variability can affect the estimates of dietary iodine intake sources. 

The literature reviews relied on PubMed searches for “UNaE”, “UNaC”, “sodium excretion”, “sodium concentration”, “UIC”, and “iodine concentration”. One reviewer was responsible for screening the titles and abstracts. We did not restrict the search to any specific dates, study types, or age groups, and relied exclusively on English language publications. We used references from reviewed papers and expert advice to identify additional sources.

## 3. Results

### 3.1. Considerations for Salt Intake Assessments 

#### 3.1.1. Classification of Salt Intake

In agreement with the WHO’s strong recommendation, the World Hypertension League (WHL) advises a daily sodium intake of less than 2000 mg (5 g salt) [3,4]. The Dietary Guidelines for Americans advise a slightly higher amount (less than 2300 mg, 5.8 g salt), but also include an additional, lower threshold for specific people: 1500 mg sodium (3.8 g salt) is recommended “among persons who are 51 and older and those of any age who are African American or have hypertension, diabetes, or chronic kidney disease”. These subgroups represent approximately 50% of the US population [5] and the 1500 mg recommendation is intended to ensure a minimum intake. 

In the US, there is no Recommended Dietary Allowance, but there is an Adequate Intake of 1520 mg sodium (3.8 g salt) “to cover sodium sweat losses in un-acclimatized individuals who are exposed to high temperatures or who become physically active” [6]. The WHL bases minimum sodium needs on ancestral levels, placing it at 1000 mg [3]. The WHO and the US do not classify intakes greater than 2000 mg, but WHL has described high, very high, and extremely high intakes as greater than 2000, 4000, and 6000 mg, respectively. The WHL individual level cutoffs and intake ranges are based on ancestral levels of salt intake and on studies that compared 24-h UNaE levels with blood pressure increase [3,6]. 

At the population level, the WHO recommends the same daily intake as for individuals, 2000 mg sodium, as the average intended for chronic disease prevention [7]. Also at the population level, the WHO Global Action Plan for the Prevention and Control of Non-Communicable Diseases 2013–2020 includes a target of 30% reduction in national, mean sodium consumption [8]. The action plan’s evaluation indicator is the age-standardized mean population intake of salt (sodium chloride) per day in grams among persons aged 18+ years.

Globally, the average national daily intake is close to 4000 mg sodium (10 g salt) [3]. A 30% reduction, recommended by the WHO, would therefore represent a decrease of 1200 mg sodium (3 g salt) of the daily intake [3]. According to the WHL recommended nomenclature for salt intake reduction, this is a moderate decrease. As with individual level cutoffs and intake ranges, the levels set to classify reductions of salt intake in populations are based on mean 24-h UNaE values.

#### 3.1.2. Measuring Salt Consumption

Dietary methods for measuring salt consumption are challenging, mostly due to their limited ability to capture the discretionary uses of salt. The amount of salt intake from salt additions during food preparation in homes, restaurants, canteens, or catering operations cannot be determined accurately with common dietary recall [9] or record methods [10]. Importantly, a large part of the salt that people consume in industrialized countries and, increasingly, also developing countries [11], is supplied through commercially manufactured foods, while food composition tables do not usually reflect changes or differences in the salt content of processed foodstuffs by manufacturer, brand, or major restaurant chain. Dietary consumption studies therefore typically underestimate the true salt intake [12,13,14]. 

In contrast, excreted urine captures the discretionary salt intake and does not rely on detailed food composition data to calculate sodium or salt intake estimates. The amount of sodium in urine collected over a timed, consecutive 24-h period (UNaE) is regarded as the gold standard by the World Health Organization [15], the Pan-American Health Organization (PAHO) [16], the US Centers for Disease Control and Prevention (CDC) [17], and the US Institute of Medicine (IOM) [18] for estimating the salt intakes of individuals and populations. Key considerations for deeming UNaE the gold standard for salt intake estimates are that UNaE accurately captures at least 90% of ingested sodium [18], and provides the best estimate of the “population profile distribution and average level of dietary salt intake [16]”. The major limitation of UNaE, however, is that the collection of all urine voids during a 24-h period places a heavy burden on the respondents and presents logistical and financial challenges in large-scale surveys. A 2015 review on measuring sodium intake [19] found that among 14 recent population studies using UNaE, the mean response rate (completed 24-h urine collection) was 38%, with values ranging from 9% to 88%. The majority of studies had a response rate below 40%, which is low enough to question the representativeness of the study respondents. Although UNaE is the accepted gold standard, the practical drawbacks may mean that other methods are superior for individual-level assessments of salt intakes, as well as for population-level classification and identification of high-risk groups. 

#### 3.1.3. Estimating Population Salt Intakes

In the US, UNaE is not included in the National Health and Nutrition Examination Survey (NHANES) due to the respondent burden and logistical challenges [18]. Reducing the 24-h collection period, for example to a 12-h or overnight collection, has been examined in some studies, but does not adequately reduce respondent burden [19] and can introduce systematic error [20]. The most apparent alternative approach to reduce respondent burden and survey costs is casual urine sampling. Studies of casual urine collections typically achieve a response rate greater than 70% [19]. The UNaC values from casual urine samples can be converted to predicted 24-h urinary sodium excretion values, i.e., daily salt intake estimates. The WHO and PAHO recommend an initial UNaE study to establish a population-specific conversion equation, and then to use the less expensive and cumbersome UNaC for ongoing surveillance [15]. Even though the US CDC recommends against using UNaC for individual estimates of salt intake, it states that UNaC can be used in monitoring variations of average population intakes [17]. There seems to be an emerging consensus among the health organizations that predicted-UNaE estimates, derived from the conversion of UNaC values, can provide comparable information to UNaE measurements for population-level monitoring and assessments of salt intake reduction.

A global assessment of monitoring systems in 2012 included 30 countries that conduct national salt intake monitoring. Of these 30 countries, four (Australia, New Zealand, Vietnam, and Ireland) reported using casual urine samples at the national level with the remainder of countries using 24-h dietary recall, 24-h sodium excretion, or a mixture of methods [21]. A review in 2015 of population-based (both national and subnational) studies of sodium intake from urine biomarkers since 2000 showed that of 29 studies, 17 collected UNaE, 8 collected UNaC, and 4 relied on overnight or 12-h urine sampling [19]. Of the 8 studies that used UNaC, 4 were national level studies in England, South Korea, Scotland, and the US. In addition to these two reviews, which identified 8 countries that have used UNaC in national surveys, we identified Germany as another country that has collected UNaC at the national level [22]. Of these 9 countries, Scotland [23] and Germany [22] reported the basic UNaC findings; the other countries reported the salt intake estimates from predicted-UNaE [18,24,25,26,27,28,29]. For all these countries, except Australia, we were able to identify the reports on the use of UNaC data to estimate the national salt intake values; the reports were published at 3–5 years after completion of the survey. The findings of these studies are shown in Table 1.

UNaC survey data can be used to estimate average population salt intakes and it should, therefore, be possible to compare the salt intake estimates between countries. The interpretation of differences in estimated salt intakes between countries is not a straightforward matter, however, because of the variable age structure of the survey participants and because of differences in conversion equations and in the reporting of results [22,23,30,31,32,34,35,36]. The bleakest methodological difference in the country data set of Table 1 is that the England and Scotland surveys report values for salt intake in the original UNaC units, i.e., without conversion to daily intakes, while each of the other surveys report intake estimates obtained from a conversion of UNaC. As expected, the reported England and Scotland values are substantially lower than the values of the other countries because they are expressed per liter as opposed to per day. For the countries that did adjust their UNaC findings to predicted-UNaE, the estimates do provide accurate average daily salt intakes, but each country elected to use a different conversion formula. As the different equations have been shown to provide different estimates [32,36,37,38], caution must be exercised when comparing the country estimates. An additional difference in analysis methodology is that the countries did not use the same measure for reporting the average intake estimate—some used the arithmetic mean, others the geometric mean, and yet others the median. Distributions that are not normally distributed, as may be the case for unadjusted UNaC values, can yield different measures of central tendency. 

While comparing countries is a challenge when methodologies differ, when countries collect salt intake estimates over time with the same methodology, the reported values would allow for time trend analysis. In the US for example, the 1988–2010 trends from converted UNaC and from 24-h dietary recalls were compared. The two methods yielded a very similar change with time: over the 12 years period, the converted UNaC values showed an increase of 130 mg while an increase of 120 mg was found from the 24-h dietary recall data [34]. This US finding offers some confirmation that UNaC can be useful in trend monitoring, but ideally the UNaC time trend should also match with findings based on the gold standard UNaE. 

**Figure 1:**
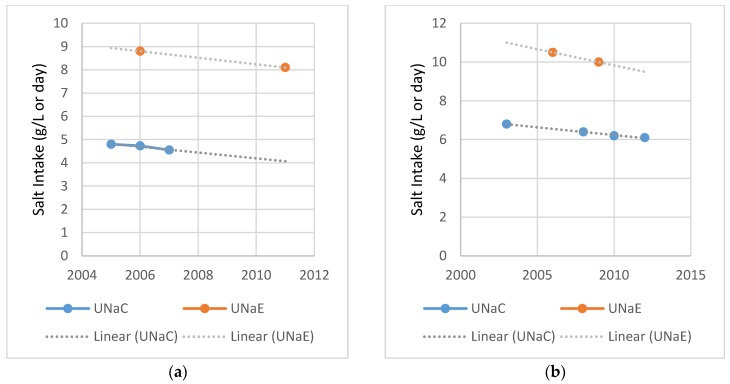
Comparison of National Salt Intake Trends Using 24-h Sodium Excretion and Urinary Spot Tests (**a**) England national salt intake estimates from UNaE and unadjusted UNaC, Millett 2012 and 2011 National Diet and Nutrition Survey (**b**) Scotland national salt intake estimates from UNaE and unadjusted UNaC, 2013 Scottish Health Survey and 2011 NDNS.

Salt intake monitoring in the UK uses both UNaC and UNaE, which allows for this comparison in the English and Scottish databases. Figure 1 compares the time trends in UNaC findings and UNaE estimates within England (left hand) and Scotland (right hand). The two measures in England and Scotland show downward trends of salt intakes [30]. As expected, the non-converted UNaC values are substantially lower than the measured UNaE. In Scotland, UNaC and UNaE show a similar decrease when viewed as a percent change. It is noted that, while the data points are few, UNaC and UNaE give a different percent change in England.

#### 3.1.4. Variability of Urinary Sodium Excretion

Salt (sodium chloride) is the main source of sodium in the diet [39]. The relative contributions to the total sodium intake by the discretionary use of salt (salt added during cooking and at the table), by salt used in commercially manufactured foods (including restaurants and catering), and by salt naturally present in foods are variable across societies. In industrialized countries, such as the US, commercially processed foods are the main source of sodium intakes [18]. In the UK, a study combining dietary and biochemical methods reported that 75% of the sodium intake came from processed foods, with 10% from naturally present salt, and 15% from discretionary uses [40]. On the other side of the spectrum, a study from rural China showed that 75% of sodium intake came from discretionary salt [41]. Differences in the discretionary salt use and in the salt from processed foods lead to large variations in salt intake, with most populations over-consuming. Research on diet during the prehistoric period showed an average intake of 680–768 mg sodium (1.7 g salt) per day, which is far less than the current global population estimate of 4000 mg sodium (10 g salt) [3,42]. 

Sodium is absorbed in the small intestine and colon at a ~99% absorption rate under normal conditions. The remaining ~1% is excreted in the feces. A 70 kg adult has ~100,000 mg of sodium in the body, of which one-third is kept in the skeletal structure and not available for metabolic exchange. Renal excretion and reabsorption of sodium with water is the primary mechanism to control body fluid balance and osmolality, and this connection to fluid volume regulation makes sodium closely linked to blood pressure. Sodium excretion and reabsorption is regulated by vascular pressure receptors, the renin-angiotensin-aldosterone system, and natriuretic peptides. Regulation maintains an extracellular fluid sodium concentration of ~3335 mg/L and an intracellular concentration of 276 mg/L [39]. In healthy individuals, changes in sodium intake are generally matched in time by changes in sodium output. However there are conditions that can lead to sodium excess or deficit in the body. Renal sodium excretion may not be sufficient in cases of excessive, rapid ingestion of salt, congestive heart failure, Conn syndrome, and renal failure, thus leading to sodium excess. Excessive sweating, chronic diarrhea, Addison disease, diabetes, diuretics, and renal disease can all lead to excessive sodium loss and sodium deficit [39].

Sodium intake varies day-to-day at the individual level depending on the types and amounts of foods consumed. The within-person variation of UNaE measurements can be three times greater than the variation between persons [19]. Large day-to-day differences in sodium excretion are physiologically normal. An analysis of INTERSALT data reported day-to-day UNaE correlations, adjusted for age and sex, ranging from 0.37 to 0.40, thus demonstrating that a single UNaE cannot be relied on (i.e., repeated tests do not give the same result) for estimating the usual salt intake of a person [43], which corroborates the recommendation from Simpson et al. in 1983 [44] that multiple 24-h urine collections are needed for an accurate estimate of an individual’s average, or habitual, intake. At the population level, the within-person variation resulting from normal fluctuations in sodium intake will not bias the estimate of a population’s average UNaE, but the inherent variability does limit the ability to obtain an accurate estimate of the population percentage of individuals with low or high intake. 

Within a population consuming a constant diet, the sodium intake varies by the amount of food consumed. Typically, higher UNaE is found among adult men compared to women, and among adults when compared to children, while the elderly typically have lower sodium excretion than young or middle-aged adults [19]. Thus, the mean population level of UNaE depends on, and can be affected by changes in, the age structure of the population. Also, seasonal changes in dietary energy consumption or the types of foods consumed can cause UNaE to fluctuate. A study among Israeli industrial workers showed that sodium intake increased 42% in the winter when compared to the summer [45]. The sodium excretion of individuals can also differ for reasons not related to intake (the causes of sodium excess and deficit listed previously) and it may be more difficult to account for these differences. 

Sodium is lost through urine, sweat, and feces. Since sodium levels in body fluids are tightly regulated, a change in the rate of sodium secretion through sweat can alter the amount of sodium excreted through urine. Sweating rate can be affected by climate and season. In a prospective study of American adults the percentage of sodium intake excreted in the urine was less in the summer [46]. In a controlled situation without excessive sweating, the normal sweat loss was estimated at 100–300 mg sodium [19], which corresponded to 2.5%–7.5% of the total intake based on an intake of 4000 mg sodium. In contrast, with intense physical activity for a couple of hours, 690–3220 mg sodium was lost through sweat [19]. Heat exposure can also affect sodium excretion. A study of adult males in the UK found that on average, 37% of the sodium intake was lost through sweat during 5 days of daytime heat exposure at 40 °C [47]. An initial exposure to heat, humidity, and physical activity in combination resulted in the largest increase in sweat sodium secretion and a corresponding decrease in urinary sodium excretion, but acclimatization reduced the effect. Still, at a high sodium intake of 4000 mg in this study, which is also the global average, the difference in sweat secretion between baseline without heat exposure and end-line after acclimatization was 1600 mg. Sweat sodium excretion remained elevated after acclimatization and at 22%, the amount of ingested sodium lost through sweat remained much higher than the 2.5%–7.5% loss without heat exposure [47]. Studies of the effect of heat exposure and exercise on sweat sodium secretion are difficult to carry out on a large scale, which is why the largest sample size identified by Cogswell et al. for such studies was 92 [19]. The US IOM identified that studies on sweat loss of sodium in broad populations was a research gap [6]. Cross-sectional data from Scotland did not find a seasonal difference in UNaE at the national level [48], but it remains possible that the seasonal change in climate, temperature, and exercise patterns meaningfully affect sweat sodium secretion and salt intake estimates from UNaE measurements in some populations. 

UNaE can also be reduced through watery diarrhea. In the case of watery diarrhea, sodium is abnormally secreted into the small intestine and lost through feces. A study among hospitalized children in Canada showed that children with dehydration from diarrhea had a mean urinary sodium concentration of 1518 mg compared to 3312 mg in a non-dehydrated control group [49]. Persistent diarrhea that is not treated can lead to sodium deficit in the body; sodium is an essential ingredient of effective rehydration solutions—the United Nations Children’s Fund (UNICEF) and WHO recommended oral rehydration solution, the standard treatment for diarrhea, contains 1725 mg/L of sodium. Individuals with treated or untreated diarrhea may, therefore, show UNaE values that do not correspond to their usual sodium intake. We could not identify any studies assessing the effect of diarrhea on population-level urinary sodium excretion or concentration.

Typically, healthy individuals have a circadian rhythm of blood pressure, where overnight blood pressure decreases by 10%–20% [50]. The drop in blood pressure is accompanied by reductions in urine volume and thus, sodium excretion. Overnight urine volume and sodium excretion usually decrease to 30% of the midday peak levels [51]. Individuals with impaired ability to achieve sodium balance—e.g., hypertensive individuals who have acquired salt-sensitivity, patients with diabetes or chronic kidney disease, or elderly persons—do not experience the normal decrease of nocturnal blood pressure, which leads to more nocturnal sodium excretion [52]. Though rare, genetic differences can also predispose individuals to salt sensitivity and disrupt the circadian rhythm in sodium excretion [6]. When the circadian rhythm is disrupted, instead of dropping by 30%, the nighttime sodium excretion can actually exceed daytime excretion [50]. Changes in circadian rhythm would not affect studies of 24-h urinary excretion, but could bias predicted-UNaE estimates when relying on spot or overnight urine collections. We could not identify studies that estimated the population prevalence of disrupted circadian rhythm of blood pressure and sodium excretion, but it is expected that in older populations, the genetically predisposed population, or in populations with high (chronic) disease prevalence, a sizable part of the population may have a disrupted sodium excretion pattern. Further complicating any attempt to quantify the impact of disrupted circadian rhythm on sodium concentration is that some blood pressure drugs, including diuretics, can restore the dipping pattern [52], as can a low sodium diet [53].

Diuretics are used to treat edema and decrease blood pressure by their action to reduce sodium reabsorption in the kidney. When not reabsorbed, sodium is excreted along with water in the urine [54]. For individuals taking diuretics, a UNaE measurement would not properly reflect the usual sodium intake because of the increase in sodium excretion. In 2003, 9% of the US adult population were reportedly using diuretics to treat hypertension [55], and the percentage may be higher at present because of a rising use of diuretics with the increase of hypertension diagnoses [56]. It is usual practice for population studies that estimate salt intake from UNaE measurements to exclude individuals taking diuretics at the stage of enrollment [34]. ACE inhibitor medication also reduces sodium reabsorption and is also a common exclusion criterion because of its effect on sodium excretion [34]. 

During pregnancy and lactation, increased calorie intake results in increased sodium intake. According to the IOM, additional sodium (~69 mg/day) is needed during pregnancy [6]. There are physiological changes during pregnancy that could both impair or enhance urinary sodium excretion and IOM cites a lack of evidence on how all of these factors may ultimately affect the expected excretion pattern during normal pregnancy [6]. Even if the urinary sodium excretion accurately reflects sodium intake during pregnancy and lactation, the sodium intake is higher than normal. Population level studies commonly exclude pregnant women because of the potential effect of pregnancy on UNaE values [34]. 

### 3.2. Use and Interpretation of Casual Urinary Sodium Concentrations 

#### 3.2.1. Estimating UNaE from UNaC

The concentration of sodium in urine depends to a major extent on urine volume. Both the volume and frequency of urination are highly variable, depending on an individual’s fluid intake and hydration status. In clinical practice, urinary sodium concentration is used alone or in comparison to the patient’s serum sodium concentration (i.e., fractional excretion of sodium) for diagnostic purposes, but this practice does not allow an estimate of daily salt intake. To estimate daily salt intake, the sodium value of a single casual urine sample must first be put into the context of the daily urine volume and sodium excretion, and thus, a casual sodium concentration cannot be used directly in comparison with the recommendations for daily salt intake of an individual. 

A simple method for converting UNaC values, adopted from research, is the use of a population’s expected creatinine excretion as a basic reference in converting the UNaC value in tandem with the creatinine concentration in the same casual urine sample to obtain predicted-UNaE [16,57,58]. Creatinine, metabolized from creatine phosphate in muscle, is produced and excreted at a constant rate because it is not reabsorbed in the kidney. Notably, however, creatinine is also derived directly from dietary protein, making its excretion co-dependent on the amount and fluctuation of the person’s recent protein consumption. Creatinine excretion also varies by age, sex, and other characteristics. Thus, sodium:creatinine ratios and generalized creatinine excretion estimates do not adequately take into account the population-specific variations between individuals, and may be particularly inappropriate for low-income countries where the dietary protein content is typically low and more variable from day to day [59]. It should be noted that the predicted-UNaE estimates derived from concurrent creatinine values in casual urines are not valid at the individual-level. The major underlying reason is the sizable day-to-day variation in urinary creatinine concentrations within persons, which results in higher dispersion of the predicted-UNaE estimates [60], compared to actually measured 24-h UNaE values. It is also noteworthy that estimations based on concurrent creatinine concentrations were shown to be biased for other analytes that exhibit diurnal variations [58]. 

The reviewed monitoring surveys (see Table 1) used various existing conversion formulas to derive predicted-UNaE values. As recommended by the WHO [16], a population-specific equation is preferred for calculating the predicted-UNaE from UNaC and a number of such conversion equations, using creatinine as an intermediary estimate, have been developed. Kawasaki et al. used specific timing (the second morning void) for the sampling of urine and incorporated age, height, and weight into gender-specific equations to estimate creatinine excretion [43]. Timed urine collections are hardly feasible in large-scale surveys, however. The International Cooperative Study on Salt, Other Factors, and Blood Pressure (INTERSALT) developed gender-specific equations to predict UNaE from casual urines without specific timing. For Japan, Tanaka et al. derived a single equation to predict 24-h creatinine excretion for both sexes [61]. For North American and European populations, Brown et al. [62] opted to not predict creatinine as an intermediary but to use the measured creatinine concentration, along with potassium concentration, BMI, age, and sex, to directly predict 24-h sodium excretion. Such direct predictions may have an advantage by accounting for circadian rhythm, but this was not assessed in the study. The various equations that were developed are now being used to analyze national surveys [34] and the predictive equation approach is being used to develop additional equations for other populations [38].

#### 3.2.2. UNaC and UNaE Individual and Population Level Correlations 

A 2012 systematic review addressed the correspondence between actually measured UNaE and predicted-UNaE derived from casual urine samples [63]. From 20 studies identified, eight directly compared the sodium concentration in single casual urines (UNaC) to the daily sodium intake from 24-h urine collections (UNaE). The eight studies used different approaches (timing of the casual urine sample, conversion formula, etc.) to derive predicted 24-h excretion (predicted-UNaE) and seven out of these eight studies concluded that spot urine samples could be used for population level monitoring [63]. The correlation coefficients in the eight studies between the casual-derived and 24-h actual sodium values ranged from 0.17 to 0.73, with an average of 0.44. While the majority of the authors of these studies (seven of eight) agreed that spot collections of urine are adequate for population level estimates, an average correlation below 0.5 seems rather weak for arriving at a general agreement that casual urine sodium concentrations can predict reliable individual-level sodium excretion. The authors of the review [63] were in agreement that the sodium data from casual urine collections cannot be used for reliable prediction of individual level 24-h sodium intakes, but that these data would nevertheless be more convenient and appropriate for monitoring changes in population sodium intakes. 

The reason that casual urine sampling is considered acceptable for population-level estimates (apart from the criteria of feasibility and convenience) is that, when used with the existing equations, the spot sodium concentration values can accurately predict the mean 24-h sodium excretion. The best evidence for this comes from a study that examined population-level mean agreement. In 2013, Brown et al. [62] applied the INTERSALT conversion equations to 29 different other Western populations. Across populations, strong correlation coefficients of 0.79 in men and 0.71 in women were found—well above the correlation values reported for individual level comparisons in the above-quoted systematic review [63]. Most importantly, the differences between the predicted and actually measured 24-h sodium excretions of −41.4 mg in men and +50.6 mg in women, respectively, confirmed high agreement between the casual urine-based population salt intake estimates and the actually measured 24-h excretion values. These differences are small enough to conclude that casual urine sodium concentrations can produce accurate mean 24-h sodium excretion estimates for Western populations. 

Notwithstanding the finding of a high overall mean agreement between the predicted and the actually measured 24-h sodium excretions, Brown et al. also reported that the difference between the mean 24-h sodium excretion values was greater than 460 mg in eight of the 29 populations, which led the authors to conclude that population-specific equations are necessary to avoid a biased mean estimate [62]. The finding that predictive equations cannot be applied to different populations without precaution was corroborated by another recent study that applied multiple predictive equations to a global population from 11 different countries and reported differences between the predicted and measured UNaE means of −859 mg and −547 mg for the INTERSALT and Kawasaki equations, respectively [64]. 

#### 3.2.3. Categorizing with UNaC and UNaE

In a study of normotensive adult American men and women aged 30–44 years with mean UNaE of 4053 mg, the intra-individual variation was 1325 mg, or 33%. The high variation led the authors to recommend at least 10 repeated UNaE measurements to accurately divide a population into tertiles of sodium intakes [65]. Similar studies corroborate the need for obtaining repeat UNaE measurements to improve the precision of the proportion estimates [66,67]. When using single UNaE values, the within-person variability will increase the lower and higher ends of the population’s frequency distribution. Since the tail ends of the UNaE distribution represent the population’s proportion of low and high intakes, the point estimates for these proportions will be biased, but the extent and direction of bias depends on the population’s average. The effect of over-dispersion on the classification at different salt intake levels can be quantified, but we are not aware of any study that has evaluated this effect by comparing the estimates of excess sodium intake between single and multiple UNaE measurements.

As is the case with sodium excretion, sodium concentration is also affected by large within-person variation. Wang et al. reported within-person variances for timed spot urines of 21%–41% of the mean, compared to variances of 16%–29% for 24-h excretions [68]. The larger variance for casual sampling is because sodium is not excreted uniformly through the day (diurnal variation). Single 24-h sodium excretion values are affected by day-to-day variations within individuals, which affect the dispersion of the UNaE and UNaC frequency distribution, as well as by intra-day variations of each individual, which affect only the dispersion of the spot sample concentrations. INTERSALT predictive equations have been shown to reduce this variation to levels that are even lower than directly measured 24-h excretions. Tanaka et al. reported that in the Japanese cohort, the standard deviation for measured UNaE values was 33%, while that for the predicted-UNaE was 21% of the mean [61]. After the UNaE distribution in Western populations was corrected for over-dispersion based on repeated collections of UNaE in a subsample, the standard deviation of predicted-UNaE estimates was still found to be lower than the standard deviation of directly measured and corrected (reduced) UNaE values [62]. It should be noted that UNaE predicted solely from creatinine concentration and excretion has shown the opposite effect, namely increased variability [36]. 

The different variability of the predicted-UNaE values may lead to bias and disagreement in classifying individuals with high or low salt intake. Furthermore, the effects from different estimation techniques on classification may be different and inconsistent over time. McLean et al. showed that measured UNaE and predicted-UNaE gave the same mean but different proportions when classifying a New Zealand population into groups consuming higher than recommended levels of sodium intake [36]. Estimations from creatinine can underestimate a proportion by up to 12 percentage points, while the INTERSALT Japan predictive equation can overestimate a proportion by up to 18 percentage points [36]. 

Population proportions derived from predicted-UNaE do not agree with those from measured UNaE because within-day variability in UNaC and conversion with predictive equations change the variance of predicted-UNaE. However, a distribution of directly measured UNaE also gives biased proportion estimates when it does not represent the usual sodium intakes of individuals. To evaluate how close the proportions from the predicted-UNaE are to their true values requires a comparison with the proportion estimates from repeated UNaE measurements. Brown et al. compared the distributions of predicted-UNaE estimates (i.e., derived from UNaC values) to the distribution of UNaE measurement values after correction for over-dispersion based on multiple 24-h urine collections in a subsample. At a classification threshold of 2300 mg, the average absolute differences in proportion estimates were 6.7 percentage points for women and 2.8 percentage points for men [62], while 7 of the 58 comparisons of UNaE distributions in Brown’s study showed a difference in proportions greater than 10 percentage points. The best evidence for accurate classification comes from the CDC National Center for Health Statistics, where Wang et al. compared the UNaE estimates in a sample of US adults derived from repeated casual urine samples to repeated, measured UNaE values in the same individuals, and found that the population distributions were similar [68]. These authors concluded that predicted-UNaE from repeated UNaC “produced population percentile estimates with relatively low biases and might be used to estimate the proportion of the population with excess dietary sodium intake with reasonable precision”. Addressing this issue of dispersion effect by repeat 24-h sampling is even more burdensome on the study respondents than a single 24-h collection. In contrast, a great advantage of casual urine sampling for this purpose would be that repeat collections are far more feasible. 

### 3.3. Use of Urinary Iodine and Sodium Concentrations for Partitioning of Iodine Intake Sources

A key consideration for the design of a salt iodine fortification strategy is determining how iodized salt arrives at the table for consumption. For planning and monitoring purposes, it is helpful to know how much of the dietary iodine intake comes from foods without iodized salt (native iodine sources), processed foods with iodized salt, and added iodized salt (table or cooking salt). Analysis of sodium and iodine concentrations from the same urine samples can provide some insight into these key sources of iodine intake, but there are differences in analyte characteristics that need to be taken into account. Haldimann et al. developed a method for partitioning iodine sources by predicting iodine intake with urinary sodium excretion in regression analysis [2]. Partitioning methods are currently being adapted for use on national, cross-sectional surveys in multiple countries that employ spot urine collections. The basic logic of the adapted partitioning methods is that by regressing UNaC on UIC (both measured per liter) and setting UNaC to zero, the amount of UIC that comes from native sources can be predicted. The remainder of UIC comes from processed food and added iodized salt in the households, which can be further partitioned by predicting UIC when no added iodized salt is used. Here, we focus on how sodium intake and UNaC/UIC variability can affect the current methods for partitioning, focusing on the effects on the first step of estimating the percent of iodine intake that comes from native or non-native source iodine. 

#### 3.3.1. Minimum Sodium Intake

Metabolic studies estimate that 394–788 mg of sodium per day is more than adequate for minimum physiological needs [69], and studies on Paleolithic nutrition suggest that before agriculture, human sodium consumption was within this range, at 768 mg per day [42]. In modern societies, sodium consumption is much higher than the minimum requirement and ancestral level; the global mean sodium intake in 2010 was estimated at 3950 mg per day [70]. The primary reason that sodium consumption increased five-fold is that salt is added in the home or to processed foods during manufacturing; added salt is the major source of sodium in the modern diet [41]. The 2010 INTERMAP study pointed out that “only a small proportion of sodium in foods is intrinsic”, [41] but not zero. In the 1990s in the USA, it was estimated that 11.6% of total sodium intake came from sodium intrinsic to foods, or native-source sodium [71]. In the UK, native-source sodium was estimated to represent 18% of total sodium intake [72]. We could not identify estimates of native-source sodium intake from a country with a low consumption of processed food. 

Estimates from the USA and UK can provide a rough estimate of native-source sodium consumption: applying the 11.6% and 18% estimates of the percent of total sodium intake from native-source to the 2010 global mean sodium intake (3950 mg/day) gives estimates of 458 and 711 mg sodium per day from native food sources. As should be expected, these estimates are within the range of sodium intake necessary for metabolic needs and are close to ancestral levels of sodium intake. A starting point for partitioning iodine intake from urinary sodium and iodine concentrations is to determine the amount of UNaC that comes from native sources. In a population consuming iodized salt, a simple regression equation that predicts UIC with UNaC set to the level of native-source sodium intake can provide an estimate of native-source iodine intake, or iodine intake without added, iodized salt. Haldimann et al. measured native-source sodium intake, but concluded that the 12% (0.4 g) contribution to total sodium intake in Switzerland was small, and they did not take “inherent” sodium into account when partitioning the iodine intake [2]. When setting UNaC to zero, Haldimann et al. concluded that 54% of dietary iodine came from iodized salt [2]. We estimate that using 0.4 g instead of zero in the Haldimann et al. study would have predicted that 48% of dietary iodine came from iodized salt, a difference of six percentage points.

#### 3.3.2. UIC and UNaC Diurnal Variation

The diurnal urinary excretion patterns of sodium and iodine are different. Sodium excretion follows a circadian rhythm that is connected to blood pressure; in healthy individuals, overnight blood pressure decreases and there is a corresponding reduction in sodium excretion [50,51]. The overnight reduction in sodium excretion was recently found to be equal in prepubescent and pubescent children, with average reduction compared to daytime values ranging from 23% to 44% [73]. For iodine excretion, diurnal variation is dependent on diet, with the lowest excretion occurring in the morning and the highest excretion overnight [74]. The circadian rhythm of increased overnight iodine excretion was found to be present equally in both children and adults [74]. A 2008 study by Vanacor et al. showed that the overnight urinary iodine concentration can be 26%–39% higher than afternoon and morning levels, respectively [75]. 

The data on urinary sodium and iodine concentrations intended for partitioning of the dietary iodine intake sources will likely come from school-based surveys and large-scale household surveys, both of which typically collect urine samples during daytime. Since sodium and iodine have opposite circadian rhythms, with sodium excretion decreasing overnight and iodine excretion increasing overnight, the results from morning/afternoon samples will be biased (relative to average daily intakes) in different directions. A 2013 study by Wang et al. confirmed the opposite circadian rhythm of sodium and iodine excretion in a single sample [76]. The difference in circadian rhythm is a potential source of bias in studies based on comparing urinary sodium and iodine concentrations. Compared to average daily intake, urinary sodium concentration from a daytime sample is a relative overestimate, while urinary iodine concentration is a relative underestimate. In the partitioning of dietary iodine intake with the use of UNaC and UIC data, these opposite effects of diurnal variation on regression analysis should be considered. We did not identify any study that quantifies the effect of opposite diurnal variation on the partitioning approach. 

#### 3.3.3. UIC and UNaC Within-Person Variability

The concentration of sodium and iodine in urine is influenced by what we eat, which changes between daily meals and from day to day. This means that spot samples and 24-h collections of urinary sodium and iodine taken at different times from a single individual will give different results due to within-person variability. One way to gauge the extent of within-person variability is to look at the ratio of within-person variance to between-person variance. For sodium, the “within:between” ratio can be up to 3.0 and is generally close to 1.0 [19,76,77], indicating as much difference within an individual as between individuals. For iodine, the “within:between” ratio is generally smaller than that of sodium. In single spot sampling, the ratios of urinary concentrations were 0.4 for iodine and 0.8 for sodium [76]. Although sodium has more variability, within-person variance for both analytes is high, and multiple urine collections are required for precise and accurate estimates of both sodium and iodine “habitual” excretions [43,63,78]. In the case of a single spot test, the high within-person variability will result in the population distribution being overdispersed; i.e., the individual spot estimates at the margins of the distribution will be lower and higher than their true, average (habitual) values. It should be noted that in populations with uniform dietary patterns, variability may not be high and overdispersion would not be an issue.

Partitioning of the iodine intake sources from urinary sodium and iodine concentrations uses regression analysis. Overdispersion of the variables in a regression presents a couple of issues for the accuracy and precision of the parameter estimates. When the dependent variable, in this case the UIC, is overdispersed, there will be an increased uncertainty of the slope estimate. The implication of an uncertain slope is not that the point estimate is biased, but that the estimates for the dietary iodine intake sources will have large confidence intervals. A simple, albeit costly, strategy to deal with this is to increase the sample size, which will help to narrow the confidence intervals around the average intake estimates. Overdispersion of the independent variable, i.e., the UNaC, can be more problematic because it results in bias of the estimated slope. Single spot urinary sodium values may give highly overdispersed sodium estimates that will bias the slope towards zero. The importance of iodized salt could be underestimated with a slope biased towards zero, because large changes in UNaC will incorrectly predict small changes in UIC. Increasing the sample size will not address this bias. The most straight forward way to deal with overdispersion of the explanatory variable to reduce or eliminate the biased slope is to collect repeated spot urine samples. 

We could not identify a study looking specifically at the number of repeated spot tests necessary to deal with bias of the slope estimate, but work on 24-h collection of urinary sodium may provide some insight. In 1979, Liu et al. found that with a single 24-h collection, the correlation coefficient between sodium and another variable would be biased by 52%, and that after four repeated 24-h collections the bias would be reduced to 25% [65]. In Liu’s study there was very high within-person variance of the UNaE; the “within:between” variability ratio was 3.2 [65]. We can assume that the number of required spot repeats depends on the amount of within-person variability and is related to the dietary variety of a particular study population. 

## 4. Discussion

### 4.1. Repeated UNaC

As general guidance, the US CDC has supported spot urine sampling for population surveillance of sodium intake, but it also included a forewarning that “questions remain about their usefulness for estimating the prevalence of excess sodium intake and their use in older adults and in children” [17]. These caveats are evidence-based and deserve careful consideration. That said, the adoption of 24-h urine collections as the gold standard for sodium intakes at the same time, and without including similar caveats for the classification bias and the limitation on representativeness due to non-response in 24-h urine collections, seems to disregard the drawbacks of the 24-h UNaE approach. Due to existing day-to-day variations of sodium excretion in individuals, the use of single 24-h collections leads to a bias of the point prevalence estimate for excess sodium intake. Some authors propose repeated 24-h collections as the gold standard for accurate sodium excess prevalence estimation. While repeated 24-h collections would overcome the misclassification drawback, it would at the same time exacerbate the problem of high non-response that typically affects even single 24-h urine studies. Irrespective of single or repeated 24-h urine collections, the error due to non-response is not random and cannot be quantified, which means that it cannot be corrected. Repeat 24-h collections with a high response rate would be the perfect gold standard, but for the practice of field surveys of populations, a different line of approach is needed in the quest for reliable national monitoring and evaluation of mean salt intakes and proportions beyond certain thresholds. 

For routine monitoring of salt intakes the US CDC, WHO, and others recommend that casual urine sodium concentration (UNaC) is collected and turned into predicted 24-h sodium excretion (UNaE) with a population-specific conversion equation. Under this scenario, for accurate processing of the UNaC data, conversion equations must be developed from a concurrent 24-h urine collection in the same population. At present, various countries are using different unique equations for converting spot UNaC data in national salt intake monitoring, but nearly all are predicting UNaE based on a single collection of UNaC (with a single subsample of UNaE). This is sufficient for estimating the mean salt intake without bias, but predicted-UNaE from single UNaC collections cannot provide an unbiased estimate for the proportion of excess intake. Thus, the manner in which UNaC recommendations are currently implemented may help to reduce the participant burden and improve the response rate, but it is not sufficient for addressing one of the major limitations of single UNaE, namely misclassification. The need remains for a practical way forward in routine monitoring without necessarily following the repeat UNaE measurement approach. 

When seeking a practical alternative, it is helpful to look at the conclusion of one of the first researchers on the development of conversion equations for casual UNaC collections. Analyzing the relationships between UNaE collections and spot UNaC data, Kawasaki et al. in 1982 wrote that, in their experience, repeated spot tests are feasible, while repeated 24-h collections are not [43]. Kawasaki emphasized the point that sodium excretion varies more day-to-day within the same individual than it does between individuals, such that a population’s sodium intake distribution cannot be uniquely defined from the underlying single casual samples, and they recommended repeat sampling as the major way forward. In Kawasaki’s view, therefore, the practical alternative to repeated 24-h urine collections is repeated casual urine sampling. The main advantage of repeat spot UNaC sampling, compared to repeat 24-h UNaE collections for addressing the classification limitation, is that repeat spot sampling from the same individual is less burdensome and cheaper. Repetition will reduce variance and lead to a more robust character of the population’s salt intake distribution, thus permitting less bias of the salt intake estimates below or above threshold levels. 

Despite the available evidence suggesting it is inappropriate, single UNaE data continue to be used to report the percentage of the population with excess salt intake. Secondary analysis to demonstrate the extent of single UNaE misclassification could help to emphasize the need for repeats. There is also a need for additional evidence on the classification agreement between repeated UNaE and predicted-UNaE derived from repeated UNaC. One study has looked at this [68], but more evidence from different populations is needed to demonstrate the extent to which repeat UNaC sampling can successfully address the misclassification from single UNaE. Such research is needed not only to quantify the value of repeated UNaC, but also to determine how many times and among how many individuals the UNaC sampling should be repeated. Repeating urinary sodium measurements reduces the error of misclassification because it permits an estimate of salt intake with reduced variation, i.e., closer to the true population variance. Another advantage of reduced variance is that the confidence intervals for the estimates of excess intake proportions are tightened. We are not aware of any study that illustrates the effect of repeat UNaC collections on sample size requirements. With repeated measurements, a smaller sample size would be required for the same precision, thereby offsetting the increase in cost from repetition. Research on sample size is needed to determine the real cost of repeat urine collections against the benefit of more accurate estimates of salt intake. 

If repeated UNaC data is to be used for the monitoring and comparing of population salt intakes, it is important to acknowledge and account for the limitations of the basic measure, many of which also apply to UNaE. Researchers must account for factors that affect salt intake and/or metabolism in the analysis of UNaC data, such as age, acute and chronic disease, pregnancy, and seasonality. Age standardization, disaggregation or exclusion, and carrying out surveys at the same time of the year are common strategies to avoid bias. Diurnal abnormality is a potential source of bias for repeated UNaC. In addition to providing information on the classification agreement, more research on individual correlation with repeated measurements can provide insight into how much predicted-UNaE is biased by abnormal circadian rhythm. Such research will provide valuable information for targeting and will provide a more balanced view of the value of UNaC.

### 4.2. Partitioning Iodine

We illustrated that ignoring native-source sodium consumption can meaningfully affect the partitioning of iodine intake sources from UIC and UNaC data in the same urine samples. The current partitioning approach does not take into account native-source sodium consumption and may thereby overestimate the contribution of iodized salt to iodine intake. In the case where native-source sodium consumption is not available, native-source UNaC can be estimated based on data from similar populations. Another approach would be a population-specific metabolic study that restricts added salt to measure UNaC basal levels, but this may not be practically feasible. Since sodium is naturally present in a variety of foods, there may not be a large difference in the native-source sodium intake between populations, and estimating the native-source UNaC based on observations from another population may therefore be a reasonable alternative. To determine if single estimates of native-source sodium intake can be used across populations for the partitioning of iodine intake sources, more research is needed in populations with different typical diets.

Statistical considerations indicate that UIC and UNaC variability can lead to bias in the current partitioning methodology. We described why the opposite circadian rhythm of sodium and iodine, and regression dilution from high UNaC variability are specific concerns. Regression dilution bias typically overestimates the native-source iodine intake, which can be corrected for by repeat UNaC collections for the entire sample or a subsample of target individuals. The latter approach was used in INTERSALT and has been repeated in other studies [62,79]. Research is needed to determine the extent of bias from single versus repeated UNaC sampling in the partitioning methodology. An alternative approach to deal with high within-person variability is to carry out the regression analysis at the population level rather than the individual level. Brown et al. used the population-level approach to look at correlations between urinary sodium concentration from spot samples and urinary sodium excretion from 24-h collections [62]. Regression analysis to partition iodine intakes with the use of the mean UNaC at the school or cluster level would likely be less affected by regression dilution bias, but the estimates would be limited to the iodine intakes coming from native and non-native food sources only. To distinguish between the amounts of iodine intake from processed food salt versus household salt, observations with no iodine in salt are needed, which is unlikely at the school or cluster level. Differentiation between the non-native sources of iodine would not be possible in such cases. Nonetheless, population-level analysis is useful to determine the effect of regression dilution bias on the first step of partitioning studies that do not have repeat UNaC data. Repeated measurements and population-level measurements of UNaC will not address the effect of circadian rhythm. We could not estimate the extent of possible bias from circadian rhythm and, therefore, we suggest research is needed to compare the partitioning results between spot urine sodium concentrations and sodium excretion data from 24-h urine collections.

## 5. Conclusions

Single UNaE collections may not be an appropriate gold standard for monitoring population-level salt intake because of misclassification. Repeated collection, in a sub-sample of individuals, of casual UNaC data would provide an immediate practical approach for routine monitoring of salt intake because it overcomes the bias in an excess salt intake estimate. Feasible and accurate methods of classifying salt intake can facilitate improved planning and combined monitoring of salt reduction and salt iodization strategies. For salt iodization, UNaC data can be coupled by regression analysis with UIC in the same urines to estimate the key iodine dietary intake fractions from the distinct supply sources. The current methodology for partitioning iodine intake can be improved by taking into account native-source sodium intake and inherent variability in the casual UNaC and UIC data. 

## Figures and Tables

**Table 1:** Characteristics of National Surveys Collecting Casual Urinary Sodium Concentrations (UNaC).

Survey	Method for Salt Intake Estimate in Main Report	Year Published	Age Group	Intake Estimates (g Salt) If Converted from UNaC Values (g Na). Arithmetic Means Unless Noted in the Methods Column	Methods
**2011–2012 Australian Health Survey**	Dietary	Not identified	-	-	-
**2003–2007 Health Survey for England [30]**	None	2012	16+ years	All adults (*n* = 1775): 2007—4.6, 2006—4.7, 2005—4.8, 2004—6.0, 2003—5.3	Not adjusted to urinary sodium excretion (UNaE), geometric mean
**2008–2011 German Health Interview and Examination Survey for Adults (DGES study) [22]**	None	2014	18–79 years	2008–2011—(m) 10.0 (*n* = 3900), (f) 8.4 (*n* = 3276)	Adjusted to UNaE with estimated 24-h creatinine, median
**2011 Ireland National Adult Nutrition Survey [31]**	Dietary	2011	18–64 years	2008–2010—(m) 11.1 (*n* = 4329), (f) 8.5 (*n* = 3315)	* Methodology not identified; appears adjusted to UNaE
**2009–2011 Korea National Health and Nutrition Examination Survey [32]**	Dietary	2014	18+ years	2009–2011 (normotensives) 9.7 (*n* = 3801), (hypertensives) 10.2 (*n* = 3967)	Adjusted to UNaE with Korean equation
**2008/9 New Zealand Adult Nutrition Survey [33]**	None	* Unpublished	15+ years	All adults (*n* = 3544): 2008/9—9.1	Adjusted to UNaE with WHO equation
**2013 Scottish Health Survey [23]**	UNaC	2014	16+ years	2012/13: 6.1 (*n* = 2394), 2010/11: 6.2, 2008/09: 6.4, 2003: 6.8	Not adjusted to UNaE
**2010 US National Health and Nutrition Examination Survey [34]**	Dietary	2014	20–59 years	2010: 8.4 (*n* = 3290), 2003–2006: 8.4, 1988–1994: 8.1	Adjusted to UNaE with International Cooperative Study on Salt, Other Factors, and Blood Pressure North America/Europe equation
**2009 Vietnam STEPwise Approach to Surveillance Survey [35]**	Not identified	* Unpublished	25–64 years	2009: (m) 10.2 (*n* = 3978), (w) 9.5 (*n* = 3705)	Adjusted to UNaE with Tanaka equation

* Unpublished New Zealand and Vietnam UNaC estimates were extracted from doctoral dissertations [33,35]; The Ireland UNaC full report could not be identified and results are from an executive summary that did not include details of the methodology [31].
